# Life-space mobility assessment in older people in Finland; measurement properties in winter and spring

**DOI:** 10.1186/1756-0500-7-323

**Published:** 2014-05-30

**Authors:** Erja Portegijs, Susanne Iwarsson, Merja Rantakokko, Anne Viljanen, Taina Rantanen

**Affiliations:** 1 Gerontology Research Center and Department of Health Sciences, University of Jyväskylä, P.O. Box 35 (viv), Jyväskylä FI-40014, Finland; 2 Department of Health Sciences, Lund University, Lund, Sweden

**Keywords:** Reliability, Scale, Methods, Psychometrics, Mobility, Life-space, Aging, Season

## Abstract

**Background:**

Life-space mobility refers to the spatial area an individual moves through, the frequency and need for assistance. Based on the assumption that measurement scale properties are context-specific, we tested the scale distribution, responsiveness, and reproducibility of the 15-item University of Alabama at Birmingham Study of Aging Life-Space Assessment in older people in Finland, specifically accounting for season.

**Methods:**

Community-dwelling older men and women in central Finland aged 75-90 years were interviewed to determine life-space mobility (score range 0-120). Baseline (January-June 2012) and one-year follow-up data (January-June 2013; n = 806) from the cohort study “Life-space mobility in old age” were used to investigate the scale distribution and responsiveness over a period of one year. In addition, with a sub-sample in conjunction with the one-year follow-up, we collected data to study the two-week test-retest reproducibility (n = 18 winter and n = 21 spring 2013).

**Results:**

The median life-space mobility score at baseline was 64. The median change in score over the one-year follow-up was zero. However, participants reporting a decline in health (repeated measures ANOVA p = .016) or mobility (p = .002) status demonstrated a significantly larger decrease in life-space mobility score than those reporting no or positive changes over the year. The two-week intra-class correlation (ICC) coefficient was .72. Lower ICC was found in the winter than in the spring sample and for items that represent higher life-space levels.

**Conclusions:**

The test-retest reproducibility of the Life-Space Assessment was fair but somewhat compromised in the winter. Mobility of older people at the life-space levels of “town” and “beyond town” may be more variable. Life-space mobility was responsive to change, regardless of season. Further study is warranted to obtain insight in the factors contributing to seasonal effects.

## Background

Life-space mobility refers to the size of the spatial area (bedroom, home, outside home, neighborhood, town, and beyond town) that an individual purposely moves through in daily life, and to the frequency of movement within a specific time and the need for assistance [1]. Thus, it reflects the actual performance of mobility activities in daily life. Importantly, life-space mobility has been associated with quality of life in older people [2].

Life-space mobility can be studied using the University of Alabama at Birmingham Study of Aging Life-Space Assessment [1]. Previous research has established the validity and reliability of the scale among relatively healthy older people in the USA [1,3,4], South American [5] populations and in powered wheel chair users in Canada [6]. It has been argued that the measurement properties of a scale are context-specific [7-9]. Thus, sample characteristics (e.g. age and health status) and for example contextual factors, such as climate and physical environment, may affect life-space mobility and consequently also the accuracy and reliability of the assessment. The present study was conducted in Finland, a Nordic country that has a climate including long, cold winters and warm summers, and rapid changes in temperature [10]. Furthermore, Finland has extensive rural areas and towns that may cover large surface areas [11].

In older people the most common physical activity is walking [12,13]. Life-space mobility is a measure that captures the outdoor activity of an individual, irrespective of the mode of transportation [1,14], Even when using motorized transportation, walking is a prerequisite for moving in larger life-space areas. A previous study demonstrated a relationship between physical activity and life-space mobility [4], and another study used life-space mobility as an indicator of physical activity [15]. Previous studies have shown that season and weather circumstances affect the level of physical activity and walking, even in countries with a temperate climate (for review see [16,17]). Extremely low or high outdoor temperatures [18-23], shorter daylight time [19,24], fewer hours of sunshine [19,20], rainfall [20-22,24], higher wind speed [20,22], and snow and icy conditions [22,25] have been identified as barriers for physical activity and walking behavior. Most previous studies have focused on children, adolescents and working-aged adults (for review see [16,17]). Studies among older people have shown similar associations and some researchers have suggested that the effects of weather may be more pronounced among older people [19,24-26]. Still, whether life-space mobility is affected by season or weather has not been studied before.

The aim of the present study was to investigate selected measurement properties of the Life-Space Assessment in community-dwelling older people in Finland. We studied the distribution, one-year responsiveness and two-week reproducibility of the scale items and composite scores. Considering the potential impact of season and outdoor temperature on outdoor behavior patterns, we also conducted stratified analyses for season (winter and spring).

## Methods

### Study design and participants

The present study was conducted as part of the “Life-space mobility in old age” (LISPE) two-year prospective cohort study of community-dwelling older people aged 75 to 90 and living in the municipalities of Muurame and Jyväskylä, located in central Finland [27]. The recruitment area spanned an area of 1660 square kilometers [11] and included urban, suburban and rural areas. Based on the 30-year average, this area is covered by snow from November to April [10]. Measurement properties of the Life-Space Assessment were studied using the baseline data, and responsiveness was studied using the baseline and one-year follow-up data. In conjunction with the one-year follow-up, two-week reproducibility of the Life-Space Assessment was studied using the data of a sub-sample.

Details of the study methods, including non-respondent analyses, have been published in a protocol paper [27]. To summarize, first, written information on the study was sent to a random sample of participants (n = 2500) drawn from the population register and then they were contacted by phone to ascertain study eligibility. Inclusion criteria of the cohort study were living independently, able to communicate, and residing in the recruitment area. Subsequently, a written informed consent form was signed during the home visit and the baseline interview was administered (n = 848, January-June 2012). All interviewers received special training before the study started. One year after the baseline data collection, which was targeted within a two-week range from the baseline date, all participants were contacted again for a phone interview that targeted the main variables of the project. If the participant was unable to communicate, a proxy (if available) was interviewed. Participants were not excluded if the two-week time range was not reached, e.g. due to illness or holidays. The LISPE project was approved by the Ethical Committee of the University of Jyväskylä, Finland.

In conjunction with the one-year follow-up phone interview in 2013, data of a sub-sample of consecutive participants (N = 41) was used to study the two-week reproducibility. Data was collected in week 9 (February; winter conditions with average temperature below zero and snow on the ground) and week 19 (May; spring conditions with higher temperatures and no snow). Two interviewers each invited 10 consecutive participants willing to participate in a retest, a second phone interview, in which two weeks later the Life-Space Assessment was re-administered by the same interviewer.

### Life-space assessment

Life-space mobility was measured with the 15-item University of Alabama at Birmingham Study of Aging Life-Space Assessment [1] that was translated in Finnish [27]. Life-space mobility reflects actual mobility performance in daily life during the 4 weeks preceding the assessment. For each life-space *level* (bedroom (score 0), other rooms (1), outside home (2), neighborhood (3), town (4), beyond town (5)), participants were asked how many days a week they attained that level (*frequency*; <1×/week (score 1), 1-3x/week (2), 4-6x/week (3), daily (4)) and whether they needed help from another person or from assistive devices (*assistance*; no assistance (score 2), equipment only (1½), personal assistance needed (1)). The following indicators of life-space were calculated: 1) *independent life-space*, indicating the highest life-space level attained without help from any devices or persons, 2) *assisted life-space* indicating the highest life-space level attained using assistive devices if needed but not the help of another person, 3) *maximal life-space*, indicating the highest life-space level attained with the help of devices and/or persons if needed, 4) a composite score of *life-space mobility* which reflects the distance, frequency and level of independence (level score * frequency score * assistance score at respective level, and then summed for all levels; range 0-120), 5) a composite score of *life-space dependency* which reflects the distance and level of independence (level score * assistance score at respective level, and then summed; range 0-30), and 6) a composite score of *life-space frequency* which reflects the distance and frequency (level score * frequency score at respective level, and then summed; range 0-60). For each life-space indicator, higher scores indicate greater life-space mobility. In addition, *limited life-space* was defined as the independent life-space being restricted to the neighborhood level or lower.

### Other variables

The demographic variables *age* and *gender* were derived from the national population register; age at the time of baseline data collection was used. The month of the baseline data collection was used to determine the *season*; winter (January to March), intermediate (April), and spring (May and June). For 95 participants (12%), the baseline and the one-year follow-up data collection occurred in different seasons, more specifically a change to or from the intermediate season. The average *outdoor temperature* over the four weeks preceding each data collection (including the week of the data collection) was calculated. Daily outdoor temperature values (the mean value of four measurement points located in different parts of the study area recorded at 1 p.m.) were obtained from the local energy company (Jyväskylän Energia Ltd). At the one-year follow-up, participants were asked with an open ended question on whether they *perceived that a change in health or mobility* had occurred during the past year. Their responses were re-coded into stable, improved or declined. Some participants reported more than one change (mostly a decline) in health (n = 101) and mobility (n = 5). Eleven participants reported both improvement and decline in health and were assigned to the “stable” group. Due to low numbers (health N = 41 and mobility N = 46), those that improved were also included in the “stable” group and thus the group was renamed “stable or improved”. Perceived change in health or mobility was considered a marker of change relevant to the participant.

### Statistical analyses

Medians and interquartile ranges (IQR) are reported for all variables unless stated otherwise. The normality of the data distribution was tested with Kolmogorov-Smirnov tests.

### Scale distribution and responsiveness

Floor and ceiling effects were considered present if more than 15% of the respondents achieved the lowest or highest possible score [7]. Baseline group differences in life-space mobility score were tested using independent T-Tests or ANOVA. The prevalence of a change of ≥10 points in life-space mobility score, that is, considered a clinically meaningful change [1], was determined over the one-year period. Responsiveness was studied using anchor-based methods [7,28]; within-group changes in life-space measures were compared using several external criteria: groups of self-reported change (declined vs. stable or improved) in health and mobility, five-year age groups, and season. Interaction effects of group and time were tested with Generalized Estimation Equation (GEE) models. Life-space mobility was assessed with a linear response, life-space dependency and frequency with a gamma log-link response, and maximal, assisted and independent life-space with an ordinal logistic response. The analyses were also performed excluding participants reporting improvement in health or mobility, however, since the results were similar, only analyses including all participants are reported. Spearman correlation coefficients were calculated to test associations between the one-year change in life-space variables, self-reported health or mobility change, five-year age-group, season, change in temperature, and baseline data collection month. The one-year within-person change in frequency and level of assistance at each life-space level was tested with McNemar tests.

### Test-retest

Data inspection revealed that for three participants the circumstances changed within the two weeks between the test and retest, due to a move to the summer cottage (n = 2) or a temporarily injury or illness (n = 1). Analyses were performed twice, once including and once excluding the participants for which the circumstances changed. However, only the analyses including all participants are reported, since both analyses rendered similar results. Furthermore, separate as well as combined analyses were conducted for the winter and spring samples.

The two-week test-retest analyses included measures of agreement and reproducibility. Percentage of agreement for categorical variables or the limits of agreement from Bland-Altman plots (displaying the change in scores against the average of scores from the test and re-test) [29] are reported. Reproducibility was tested with intra-class correlation (ICC) coefficients for agreement or Cohen’s Kappa, and the 95% confidence intervals (CI) were calculated. Missing responses on the items of level of assistance and frequency at each life-space level were re-coded to include a category “not at this level”. These new variables were used for the itemized analyses of reproducibility.

IBM SPSS Statistics 20 was used for statistical analyses, and statistical significance was set at P < .05.

## Results

### Participant and contextual characteristics

As previously published [2], at baseline the participants were on average 80.6 years old (±standard deviation 4.2). Of the 848 baseline participants, 808 participated in the follow-up one year later: Reasons for non-participation in the follow-up were: died n = 16, institutional care n = 4, moved outside of study area n = 2, unable to contact n = 3, poor health n = 5, unable to communicate n = 6, not willing n = 3, and data lost due to technical problem n = 1. The life-space data were self-reported by 806 participants and for 2 people proxy reports were received. Only the self-report data were used in the present analyses. The average number of days between the assessments was 362 ± SD 9.3. At baseline, the range of outdoor temperatures (January-June) was -25.7°C to 23.0°C and at the one-year follow-up -22.7°C to 26.6°C.

### Scale distribution and responsiveness

**Figure 1:**
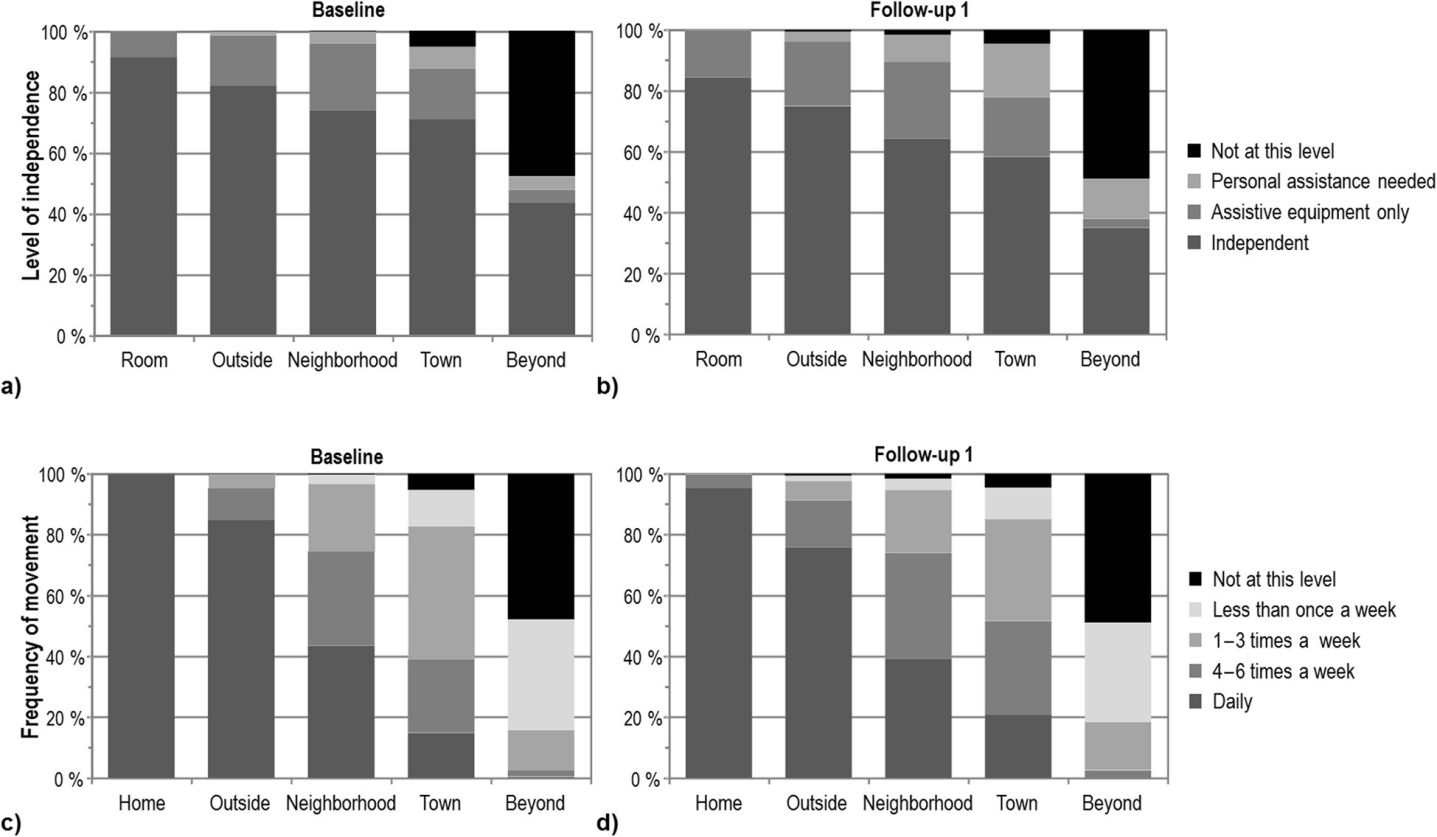
The assistance needed (a, b) and frequency of movement (c, d) at each life-space level (n = 806).

At baseline, 52% (n = 421) of the participants reached the highest possible level for maximal life-space, 44% (n = 352) for independent life-space and 48% (n = 388) for assisted life-space, respectively. For the composite scores, 42% (n = 340) obtained the highest possible score for life-space dependency, while <1% obtained the highest possible life-space mobility and frequency scores, respectively. Life-space mobility score was normally distributed.At baseline and the one-year follow-up, 62% (n = 499) of the participants reported reaching the same life-space level. Figure 1a and b show that in general the assistance needed at each life-space level increased over the one-year follow-up (McNemar p < .001). The frequency decreased significantly at the “outside home” and “neighborhood” level (p = .001), while it increased at the “town” level (p < .001; Figure 1c and d).

The median change in life-space mobility score between baseline and the one-year follow-up was zero (IQR 21; Table 1). However, 33% of the participants experienced a decrease and 24% an increase of ≥10 points between the baseline and one-year follow-up. Table 2 shows that a self-reported decline in health (p = .016) or mobility (p = .002) over the year and older age (p = .011) were associated with larger decreases in life-space mobility score. Similarly, a more restricted assisted life-space was associated with a decline in health (p = .007), mobility (p < .001), and older age (p = .004), and a more restricted independent life-space with a mobility decline only (p = .003). Changes in life-space dependency, life-space frequency, maximal life-space, and limited life-space were unrelated to self-reported changes in health or mobility or age-group. The season in which the data collection took place did not affect the change in any life-space measure. However, at baseline the life-space mobility (ANOVA p = .001), dependency (p < .001) and frequency (p < .001) scores were higher in those assessed in spring than in those assessed in winter. Further, a weak but statistically significant association (Spearman R = .120, p = .001) was found between the change in life-space mobility score and the outdoor temperature difference between the baseline and one-year follow-up. However, the change in life-space mobility score over the year was independent of the baseline data collection month (R = .000, p = .992).

**Table 1:** Indicators of the life-space assessment in participants included in the responsiveness and reproducibility samples

	**Baseline (n = 806)**		**Change to follow-up 1 (n = 806)**			**Test (n = 39)**		**Change to retest (n = 39)**		
	**Median**^**†**^	**IQR**^**‡**^	**Median**	**(IQR)**	**Range**	**Median**^**†**^	**IQR**^**‡**^	**Median**	**(IQR)**	**Range**
**Life-space mobility (p)**	64	30	0	21	-66 – 84	56.0	26.0	0	16.0	-40 – 40
**Life-space dependency (p)**	20.5	10	0	5	-21 – 18	20.0	10.0	0	0.5	-18 – 10
**Life-space frequency (p)**	34	13	0	11	-29 – 42	29.0	11.0	0	8.0	-20 – 20
**Maximal life-space (%)**										
Beyond town	52	421	0	0	-4 – 2	41	16	0	0	-2 – 1
Town	43	343				46	18			
Neighborhood	5	42				13	5			
**Assisted life-space (%)**										
Beyond town	48	388	0	1	-5 – 4	36	14	0	0	-3 – 1
Town	38	305				46	18			
Neighborhood	14	113				18	7			
**Independent life-space (%)**										
Beyond town	44	352	0	1	-5 – 5	31	12	0	0	-3 – 1
Town	28	228				44	17			
Neighborhood	28	226				26	10			
**Limited life-space (%)**										
Yes	28	226	0	0	-1 – 1	31	12	0	0	-1 – 1
No	72	580				69	27			

IQR = Interquartile range, p = points.

^†^% for ordinal variables.

^‡^n for ordinal variables.

**Table 2:** One-year responsiveness of the life-space mobility score according to external criteria (n = 806)

		**Baseline**				**Change**					Spearman*	
		**n**	**Median**	**IQR**	**P**^**†**^	**Median**	**IQR**	**Lower limit**	**Upper limit**	**P**^**‡**^	**R**	**P**
**Criterion health**	Declined	388	60	28.9	<.001	-1.5	22	-66	84	.016	-.223	<.001
	Stable or improved	418	72	30		0	20	-52	46			
**Criterion mobility**	Declined	370	60	30	<.001	-2.5	22.4	-54	84	.002	-.217	<.001
	Stable or improved	436	72	28		0	20	-66	46			
**Age-group**	75-79	339	74	24	<.001	0	24	-53	84	.015	-.373	<.001
	80-84	270	64	27		0.3	20.5	-66	40			
	85+	197	54	26		-4	20	-54	43			
**Season**	Jan-Mar	400	62	28	.001	0	21.9	-66	43	.158	.124	<.001
	Apr	148	66	28		0	21	-46	84			
	May-Jun	258	68	30		-2	21.6	-54	46			

IQR = Interquartile range.

^†^Group difference at baseline ANOVA or *T*-test.

^‡^Generalized Estimation Equation (GEE) models, group*time interaction effect.

*Correlation coefficient between the respective variable and life-space mobility score.

### Test-retest

At the time of the one-year follow-up, 41 participants agreed to participate in the retest of the Life-Space Assessment. However, we were unable to contact two participants for the retest. Thus, the repeatability analyses were performed with data on 39 participants, 18 (6 men and 12 women) in winter circumstances and 21 (5 men and 16 women) in spring circumstances. On average the participants were re-assessed after 14 days (range 12–15). At baseline their median age was 79.2 (IQR 5.3) years. The mean temperature over the 4-week period preceding the first winter data collection (test) was -3.4 ± SD 4.5°C and at the retest -4.4 ± 5.3°C. The mean temperature for the spring data collection was 9.0 ± 4.3°C and at the retest 14.6 ± 4.9°C.

**Figure 2:**
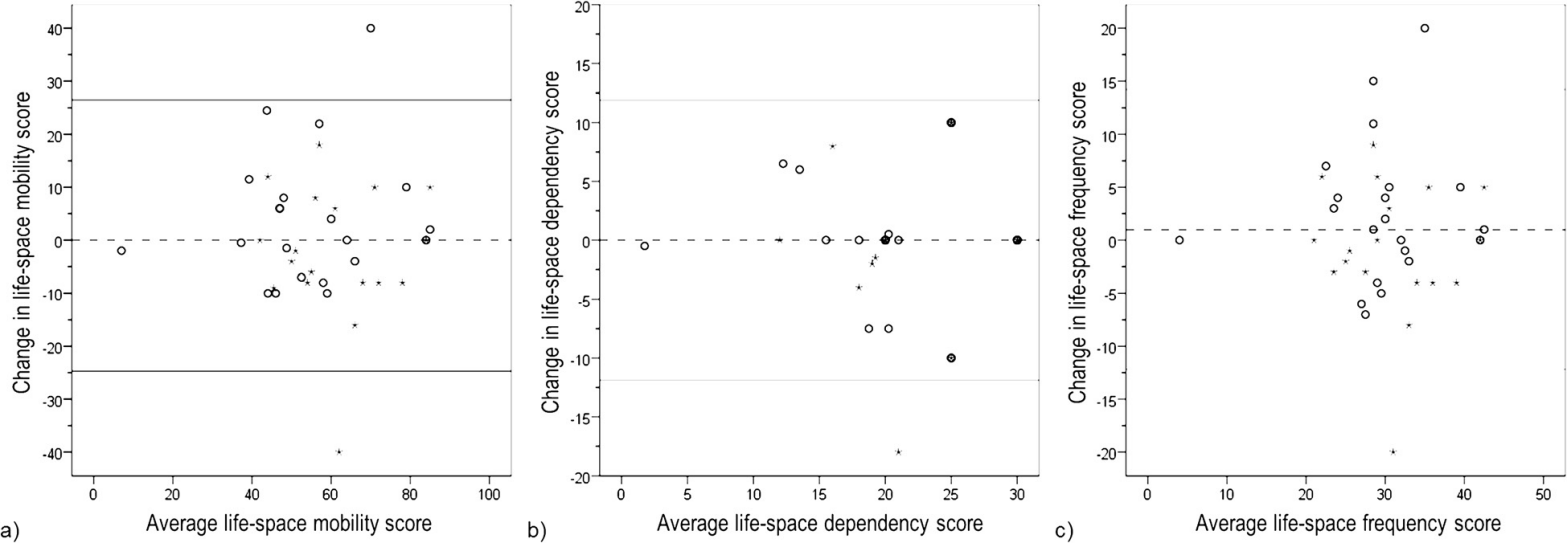
**Bland-Altman plots of the life-space mobility (a), life-space dependency (b) and life-space frequency (c) scores.** Stars represent individual participants assessed in winter and open circles those assessed in spring. The horizontal lines represent the 95% confidence intervals around the average change between the test and retest (intermittent line).

The median life-space mobility score in the winter and spring sample together at the one-year follow-up was 56 (IQR 26) points and at the retest two weeks later 54 points (IQR 20; Table 1). Over the two weeks, the median change was zero with a range from -40 to 40 points; 13% of the participants experienced an increase and 23% a decrease of ≥10 points in the life-space mobility score. The median life-space dependency score was 20 and the life-space frequency score 29 points; the two-week median changes were zero. Figure 2 shows that the change in scores was independent of the average score of life-space mobility, dependency and frequency scores, respectively.

The ICC for agreement for the life-space mobility score was .72 and slightly higher in the spring sample than in the winter sample (Table 3). The ICC for the life-space dependency and frequency score were .61 and .64, respectively. The percentage of agreement for maximal life-space, assisted life-space and independent life-space ranged between 59% and 62%, and the ICC for agreement ranged between .65 and .86. The percentage of agreement for the dichotomous variable limited life-space was 87% and the Kappa value was .69. In the winter sample, poor agreement (ICC agreement < .50) was found for life-space dependency, maximal life-space, assisted life-space, independent life-space, and limited life-space, respectively. Kappa and ICC coefficients for separate items of the Life-Space Assessment ranged between .64 and 1.0 for the life-space levels up to the neighborhood, and ranged between .33 and .62 for the life-space levels “town” and “beyond town” (Additional file 1).

**Table 3:** Percentage of agreement and Intra-Class Correlation (ICC) for the life-space indicators over the 2-week interval

	**Overall (n = 39)**			**Winter (n = 18)**		**Spring (n = 21)**	
	**% of agreement**	**ICC**	**95% CI**	**% of agreement**	**ICC**	**% of agreement**	**ICC**
Life-space mobility	-	.72***	(.52 - .84)	-	.61**	-	.76***
Life-space dependency	-	.61***	(.37 - .78)	-	.47*	-	.70***
Life-space frequency	-	.64***	(.42 - .80)	-	.60**	-	.68***
Maximal life-space	62	.65***	(.43 - .80)	72	.45*	52	.74***
Assisted life-space	59	.72***	(.53 - .84)	67	.44*	52	.78***
Independent life-space	62	.86***	(.75 - .92)	61	.43*	62	.89***
Limited life-space^†^	87	.69***	(.45 - .94)	78	.22	95	.90***

95% CI = 95% confidence interval.

^†^Kappa and 95% CI computed with Kappa ± (1.96*standard deviation).

*< .05 **< .01 ***< .001.

## Discussion

This study assesses the measurement properties of the Life-Space Assessment in a Nordic country and is the first study specifically accounting for the effects of season. The test-retest reproducibility of the Life-Space Assessment was fair, however, it seemed to be somewhat compromised in winter conditions, with average temperatures below zero and snow on the ground. In addition, the scoring of items at higher life-space levels was more variable, which may reflect the daily reality of community-dwelling older people. Further, self-reported decline in health or mobility status and age over 85 were associated with larger decreases in life-space mobility score over the one-year follow-up period, thus indicating that the Life-Space Assessment was responsive to changes.

In accordance with previous studies [1,5], life-space mobility score had a normal distribution, while the indicators of maximal, assisted and independent life-space and the life-space dependency score had ceiling effects. Also we found that the life-space mobility score and assisted life-space were responsive to change [1] as indicated by a larger change in participants reporting declines in health or mobility status over one year. The overall change in life-space mobility score related to declines in health or mobility was relatively small (1.5-2.5 points) in these community-dwelling older people compared to other studies. In clinical samples changes of 22-23 points have been reported due to surgery-related hospital admissions [30] or gynecological oncology [31], changes of nine points for non-surgical hospital admissions [30], and changes of twelve points for urogynecological surgery [31], respectively. On theoretical basis, Baker et al. [1] suggested that a change of ≥10 points can be considered clinically meaningful. In our population-based sample, about half of the participants reported such a decrease or increase between baseline and the one-year follow-up. This percentage is similar to the 6-month change reported by Baker et al. [1].

While individual change in life-space mobility and other life-space measures over the year varied, the median change was zero. It is known that community-dwelling older people experience different trajectories of declines and recovery in function [32]. A previous study of our group showed that life-space mobility scores in each 5-year age-group of advancing age were on average 10 points lower [33], the annual change in the present study did not reach the level needed to be considered clinically meaningful [1] in any age-group. However, the decrease in life-space mobility score accelerated in those aged 85 years and above. Further, individual decreases and increases of more than 10 points occurred in all age-groups. Consequently, recovery from restrictions in the life-space seems possible even in those above 85 years.

In general, the test-retest reproducibility reported in the present study was comparable to that reported by Curcio et al. [5] and Auger et al. [6], but lower than that reported by Baker et al. [1]. Also, the reproducibility of the life-space mobility scores was somewhat higher than the reproducibility of the other indicators of life-space [1,5,6]. However, in the two-week period between the test and retest, about one-third of the participants experienced a clinically meaningful change of ≥10 points [1] in positive or negative direction, which could not be attributed to a self-reported change in their situation. Similarly as for other questionnaires that assess behavior, such as self-report questionnaires on physical activity, some variation will be due to the normal variation in daily life [34]. More specifically concerning life-space mobility, previous studies have suggested that trips over longer distances occur occasionally. In older people over the age of 65 long-distance trips may occur even less than once a month [35] and may thus not always be captured within the time interval of the Life-Space Assessment.

One possible explanation of the fact that the agreement between the test and retest was slightly poorer in the winter sample for the majority of measures could be that extreme temperatures and climatologic circumstances (such as slippery roads) in the winter may affect outdoor mobility of older people [20,22,24,25]. Although in our study, the 4-weeks average temperatures for the test and retest were not different, in the winter the variation in life-space mobility may increase due to snowfall or icy conditions [22,25]. Unfortunately, such data were not available in our study.

In accordance with our expectations that were based on previous literature on physical activity and walking [19,20,22,24,26,36], life-space mobility scores were somewhat higher in spring. In spring, we observed some ambiguity in defining the “home” of some individuals for which life-space was assessed. Many Finnish people own second homes or summer cottages often in another town, and they may live in their city home in winter and all summer in their cottage in a rural region [37,38]. In the present study, we defined as the “home” of the participant their primary home in case of frequent visits for few days or a week at the time, and their secondary home in case of long-term stay (e.g. whole summer). The rating of life-space was experienced as difficult particularly in periods of transition between the different “homes”, which occur most often in the spring (and fall) [37,38]. Furthermore the use of a second home most likely affects the frequency of movement. This may be reflected in the finding that the variability in frequency at the “town” and “beyond town” level was larger in the spring sample than in the winter. In the test-retest sample two participants moved from the winter home to the summer cottage between the test and the retest, reflecting the daily reality of the LISPE study participants. Naturally, this may also affect longitudinal changes for example between baseline and follow-up data. Administering follow-up interviews on a date as close as possible to the date of a baseline interview, may not guarantee comparable circumstances since climatologic circumstances may differ regardless. Therefore we suggest that climatologic circumstances are an important factor to consider in future longitudinal studies on outdoor mobility behavior of older individuals, and that data collection should include variables able to capture such information.

Anecdotally, in the present study the interviewers and participants identified certain other difficulties when completing the Life-Space Assessment that may explain part of the variation in scoring. For example, defining the neighborhood and town area appeared sometimes difficult. In Finland, in the past decades many towns have been merged to cover extensive areas. Without structurally providing a clear-cut definition of the distance related to the town area, participants may have applied different interpretations of what area to relate to when responding. Moreover, participants living close to town borders found the use of the term “town” confusing. Further, the definition of the neighborhood was also dependent on interpretation, particularly when living in a rural area. While a distance was occasionally provided in case of ambiguity, as suggested by Baker et al. [1] and Auger et al. [6], it is unknown how other participants have interpreted the life-space areas. Consequently, inter-individual differences in interpretation may have contributed to the variation demonstrated in the results, particularly at higher life-space levels. In the original publication by Baker et al. [1], the distance perceived as neighborhood and town remained constant among study participants. The consensus distance that defined the town area [1] reported in the Life-Space Assessment manual (16 km; 10 miles) is much shorter than the distance potentially travelled within study areas such as Jyväskylä municipality in Finland. Further study is needed to determine whether the distances related to the respective life-space levels are valid in different national contexts, for example in the Nordic countries. Although this requires further study, we speculate that clear-cut definitions of the distances routinely stated in conjunction with each life-space level should be considered in future studies.

Limitations of our study include that our population-based sample consisted of relatively healthy older people, which reduced the variation in life-space mobility at the lower life-space levels. In addition, when interpreting the results of the reproducibility study, it should be taken into account that we used a relatively small convenience sample. In the responsiveness analyses, selective loss to follow-up due to declining health – a common issue in aging research – may have led to underestimation of the decrease in life-space mobility. Finally, inter-individual differences in the interpretation of the life-space areas and intra-individual transitions between different homes may have slightly contributed to the variation in results.

## Conclusions

Testing the Life-Space Assessment in a Nordic country demonstrated that the life-space mobility score was responsive to change. The test-retest reproducibility was fair but subject to seasonal variation, that is, the reproducibility was somewhat compromised in winter conditions. Further study is warranted to obtain insight in the factors contributing to seasonal effects in life-space mobility. The present study also showed that the mobility of older people at life-space levels of “town” and “beyond town” may be variable. We speculate that this likely reflects the real life situation rather than results from a reproducibility problem, but should however, be confirmed in further studies. Finally, researchers should be aware of the challenges when using the Life-Space Assessment in different contexts. Structurally defining distances for the different life-space levels may be helpful to improve reproducibility, but this requires further study.

## Abbreviations

LISPE: “Life-space mobility in old age” study; IQR: Interquartile range; ICC: Intra-class correlation; CI: Confidence interval.

## Competing interests

The authors have no competing interests to declare.

## Authors’ contributions

EP contributed to the conception, design, and data collection of the study, analysis and interpretation of the data, and writing of the article. SI contributed to the conception and design of the study, interpretation of the data, and critical revision of the article. MR contributed to the conception, design, interpretation of the data, and data collection of the study, and critical revision of the article. AV contributed to the conception and design of the study, interpretation of the data, and critical revision of the article. TR contributed to the conception, design, and data collection, interpretation of the data, and critical revision of the article as PI for the LISPE project. All the authors approved the final manuscript.

## Supplementary Material

Additional file 1: Table S4 Two-week percentage of agreement and Intra-Class Correlation (ICC) for individual Life-Space Assessment items (N=39). Click here for file
