# Supplementary material for: Life-space mobility assessment in older people in Finland; measurement properties in winter and spring
Source: BMC Res Notes. 2014 May 30;7:323. doi: 10.1186/1756-0500-7-323 (PMC4055210; doi:10.1186/1756-0500-7-323)
Supplement: Additional file 1: Table S4 — Two-week percentage of agreement and Intra-Class Correlation (ICC) for individual Life-Space Assessment items (N=39). [file 1756-0500-7-323-S1.pdf]

Additional file

Additional file 1: Table S4. Two-week percentage of agreement and Intra-Class Correlation (ICC) for individual Life-Space Assessment items (N=39).

	% of agreement	ICC	95% confidence interval
Home – level†	100	NA	-
Home – frequency	100	NA	-
Home – assistance†	95	.64***	(.18 - 1.0)
Outside home – level†	100	1***	-
Outside home – frequency	79	.80	(.66 - .89)
Outside home – assistance	97	.97***	(.95 - .99)
Neighborhood – level†	100	1***	-
Neighborhood – frequency	54	.67***	(.45 - .81)
Neighborhood – assistance	95	.95***	(.91 - .97)
Town – level†	90	.45**	(.00 - .90)
Town – frequency	53	.33*	(.03 - .58)
Town – assistance	82	.55***	(.29 - .74)
Beyond town – level†	69	.36*	(.07 - .66)
Beyond town – frequency	67	.62***	(.38 - .78)
Beyond town – assistance	69	.38**	(.07 - .62)

† Kappa and 95% confidence interval computed with Kappa±(1.96*standard deviation)

* <.05 ** <.01 *** <.001
